# Quantification of Microbial Phenotypes

**DOI:** 10.3390/metabo6040045

**Published:** 2016-12-09

**Authors:** Verónica S. Martínez, Jens O. Krömer

**Affiliations:** 1 Australian Institute for Bioengineering and Nanotechnology (AIBN), The University of Queensland, Brisbane 4072, Australia; v.salazar@uq.edu.au; 2 Centre for Microbial Electrochemical Systems (CEMES), The University of Queensland, Brisbane 4072, Australia; 3 Advanced Water Management Centre (AWMC), The University of Queensland, Brisbane 4072, Australia

**Keywords:** metabolomics, ^13^C fluxomics, thermodynamics-based network analysis

## Abstract

Metabolite profiling technologies have improved to generate close to quantitative metabolomics data, which can be employed to quantitatively describe the metabolic phenotype of an organism. Here, we review the current technologies available for quantitative metabolomics, present their advantages and drawbacks, and the current challenges to generate fully quantitative metabolomics data. Metabolomics data can be integrated into metabolic networks using thermodynamic principles to constrain the directionality of reactions. Here we explain how to estimate Gibbs energy under physiological conditions, including examples of the estimations, and the different methods for thermodynamics-based network analysis. The fundamentals of the methods and how to perform the analyses are described. Finally, an example applying quantitative metabolomics to a yeast model by ^13^C fluxomics and thermodynamics-based network analysis is presented. The example shows that (1) these two methods are complementary to each other; and (2) there is a need to take into account Gibbs energy errors. Better estimations of metabolic phenotypes will be obtained when further constraints are included in the analysis.

## 1. Introduction

In recent years, the field of microbial metabolomics has expanded dramatically. This has been mainly due to improvements in analytical platforms, such as mass spectrometry (MS) and nuclear magnetic resonance (NMR), and a more widespread accessibility to these technologies in the research community. While firstly developed for the analysis of plants [1], metabolomics quickly spread to the microbial world [2], with a main focus around applied microbiology and metabolic engineering. Unlike plants, however, microbes can be rapidly and reproducibly cultured at a large scale, making sufficient biological replication easier to achieve, and also making high quality samples more readily available for metabolite analysis.

Unlike plant tissue, microbial metabolomics faces two major problems. Firstly, the cells often need to be separated from a complex culture broth prior to analysis. This is a major challenge since absolute metabolite amounts inside the cells are often multiple orders of magnitude smaller than the respective amounts outside the cells. This means that the smallest carryover from the separation process may bias the measured intracellular concentrations. The second problem is the rapid growth of microbes and their optimization for fast turnover of substrates. This means that metabolism needs to be efficiently stopped (quenched) in a matter of seconds [3,4]. It has been shown that, for many bacteria, this is a major problem [5,6].

The most common procedure for quenching involves rapid cooling to temperatures below −20 °C which, for many different bacteria, leads to leakage of the cytoplasm into the extracellular solution [5,6]. This means that once the cells are separated from the matrix the leaked metabolites will be removed from the subsequent analysis, thereby yielding biased intra- and extracellular metabolomic profiles.

Overcoming the hurdles of sampling mentioned above enables further advancement of metabolomics to a more quantitative approach. Given the original definition of metabolomics as a non-targeted analysis of all metabolites [7], the term quantitative metabolomics is somewhat paradoxical since, to date, quantitative analysis remains a targeted approach. Nevertheless, putting semantics aside, metabolite profiling techniques continue being further developed into quantitative approaches, especially when they are embedded into a systems bio(techno)logical approach.

**Figure 1:**
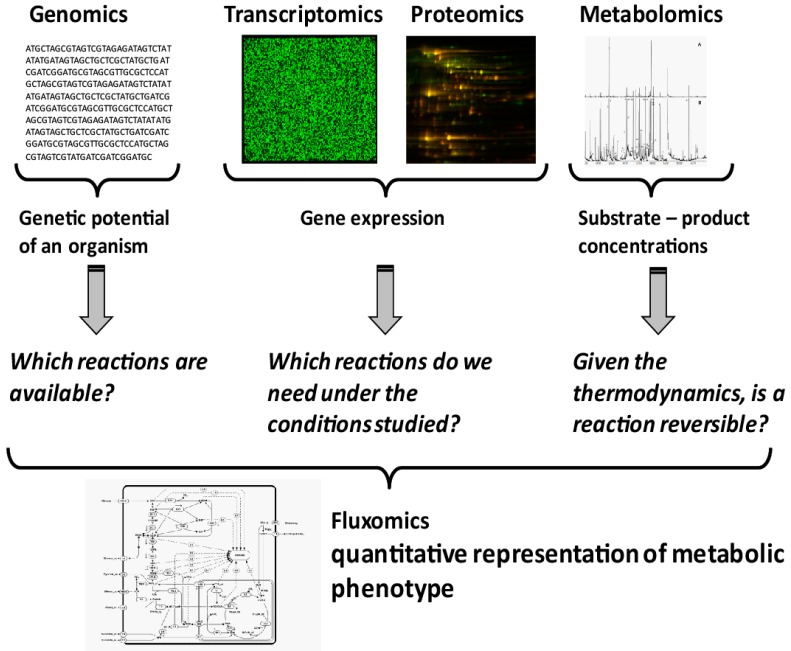
Information content in different layers of a systems biology approach.

Microbial systems biotechnology is systems biology with an applied focus [8]. Rather than just characterizing a system, a systematic understanding of the metabolic phenotypes is sought, identifying engineering targets to improve the metabolic phenotype of the cell, e.g., production of target compounds. Quantification of a metabolic phenotype is not a trivial task. It requires the integration of different datasets, as well as the creation of a model of the organism under study. While genomics provides information on the reactions that are genetically encoded in an organism (Figure 1), transcriptomics and proteomics indicate which of the encoded genes are currently expressed. This, however, gives no proof that the respective enzymes are catalytically active under the conditions studied. Due to the lack of in vivo kinetic parameters for most enzymes, the prediction of activity, or flux through a reaction, based on metabolomics and proteomics remains impossible. However, with the development of quantitative metabolite analysis, a thermodynamic modelling approach is now available. Thermodynamics provides information on the direction in which a reversible reaction will operate under the given conditions. It can also be used to evaluate whether a metabolite analysis result is thermodynamically feasible according to a metabolic model and, hence, a realistic measurement set. It is important to notice that all active reactions constraints are taken into account. Therefore, the approach can also be used to evaluate the consistency between measurements and the assumed active network. Quantifying the actual reaction rates inside cells, the metabolic phenotype, is only possible with the help of metabolic flux analysis or fluxomics (Figure 1).

While thermodynamics relies on accurate measurements of intra- and extracellular metabolite concentrations, fluxomics relies on the quantitative measurements of only a few substrates being taken up and products being secreted by the cells, as well as growth and biomass composition. When ^13^C fluxomics is performed, labelling enrichment data for metabolites is acquired using MS or NMR. This review will describe recent developments for the quantitative analysis of metabolites in microbial experiments and will then explain how a thermodynamic approach can be implemented and used to constrain the solution space for flux analysis. Finally, metabolic flux analysis principles, especially ^13^C fluxomics, will be introduced.

## 2. Quantitative Analysis of Microbial Metabolites

Several analytical platforms are available for the quantitative analysis of metabolites from microbial origins. Here, some of the most commonly used platforms will be presented.

### 2.1. High-Performance Liquid Chromatography (HPLC)

HPLC remains a workhorse technology for the quantification of metabolites, with a broad range of available columns and detectors enabling the targeted analysis of many compound classes. Its advantage over other platforms is the robustness of the methods and the availability of many detectors which are compatible with buffers or salt gradients which are unsuitable for mass spectrometry-based applications. However, sensitivity and selectivity can be an issue with HPLC. These depend on the chromatography applied, the detection method and the sample matrix. Derivatized amino acids, for example, can be detected at very low fluorescence levels (picomoles injected) with good selectivity. Another problem is that separate instrument runs are usually required for each compound class and, since identification relies on the retention times of external standards rather than mass fragmentation patterns or chemical shifts, this bears the risk of false positives. Some examples of commonly used methods are amino acid analysis [9], organic acids and sugars [10], and nucleotides and sugar phosphates [11].

### 2.2. Gas Chromatography-Mass Spectrometry (GC-MS)

GC-MS in metabolomics is mainly applied as a non-targeted semi-quantitative profiling tool, but it is a powerful tool for the quantification of volatile compounds. Quantification of non-volatile, polar compounds (after derivatization) is more complicated, since the derivatization chemistry is very matrix-dependent and the concentration of different compounds influences the derivatization efficiency of others. Each compound usually has its own reaction kinetics with the derivatization reagent [12], making determination of absolute concentrations of many compounds in a single run difficult.

For ^13^C fluxomics, however, GC-MS is currently the instrument of choice, delivering highly reproducible mass isotopomer distributions with the relative error of technical repeat injections often under 0.5%. This is possible because derivatization of the target compounds (e.g., amino acids [13]) creates stable derivatives of high molecular mass. This leads to the target ions to be moved into a mass range where fewer ions will interfere with the analysis. This results in very good signal-to-noise ratios. Another advantage is the creation of singly-charged ions and no salt adducts, leading to simpler ion chromatograms. In addition, some derivatization reagents lead to very stable fragmentation patterns, which can be used for identification purposes using databases such as NIST. Furthermore, the molecular formula of many fragments has been elucidated, a prerequisite for labelling analysis.

### 2.3. Liquid Chromatography Electro-Spray Ionization Tandem Mass Spectrometry (LC-ESI-MS/MS)

LC-ESI-MS/MS technology is becoming increasingly important for the analysis of metabolites. The advantage of this approach is its sensitivity and selectivity when run in selected (SRM) or multiple (MRM) reaction monitoring mode on a triple quadrupole instrument. These modes select a target parent ion in quadrupole 1, fragment it in the collision cell (quadrupole 2), and then measure the abundance of the daughter ion selected in quadrupole 3. The idea is that the parent-daughter relationship is very specific, enabling the filtering of a small peak among a vast pool of compounds and achieving high sensitivity when quantifying in the presence of a very small background. For example, LC-ESI-MS/MS in combination with ion-pairing chromatography was used to quantify metabolites of the central carbon metabolism [14].

The major problem with LC-ESI-MS/MS is ion suppression or enhancement during ionization [15]. This means that in certain sample matrices more or fewer ions are produced from the same amount of molecules of a compound leaving the LC column, compared to those ions formed from a known standard that is not in the same matrix. This means that many compounds will be over- or underdetermined and, sometimes, may disappear completely from the analysis. This has major practical implications on the applicability of the method: if a compound completely disappears, sample preparation or a change in chromatography may become necessary. If compound quantification is biased by matrix effects, the addition of standards may be the only reliable way to determine the true concentration of a compound. This is, of course, very labour intensive and only feasible in cases where only a few compounds are analyzed, e.g., drug metabolism studies.

The second problem with LC-ESI-MS/MS is its inherently low dynamic range. In order to get quantitative data for a microbial extract it is not uncommon to run each sample in a whole range of dilutions, especially when trying to analyze a broad range of metabolites that usually cover multiple orders of magnitude in concentration.

Another problem, that affects all analytical platforms, can be the availability of metabolite standards. For many intracellular metabolites, calibration standards may be not available off the shelf and need to be custom synthesized, adding significantly to the analysis cost, while other metabolites might not be available at all.

A recent development is the use of ^13^C labelled internal standards to overcome the ion suppression/enhancement effects caused by the sample matrix [16,17,18]. This clever approach uses the fact that many microbes can be cultured under defined conditions with the use of one, or a few, fully ^13^C labeled carbon sources. One of the cheapest labelled carbon sources is universally ^13^C labelled glucose (U^13^C glucose), which contains the stable isotope ^13^C in all carbon positions. Fortunately, glucose is also the preferred carbon source for a long list of microbes as their exclusive carbon and energy source.

When creating internal standards for all metabolites, cells are cultured under fully-labelled conditions, which mean that after achieving isotopic steady state all metabolites in a cell will have all their carbons labelled. There is a need to consider residual unlabelled carbon, which can come from the biomass originally used as the starter culture in the labelling experiment, impurities in the fully-labelled substrate (usually >99% ^13^C) and, in the case of aerobic experiments, unlabeled atmospheric CO_2_ that can enter the culture and can be fixed in carboxylation reactions. This can be established by determining the labelling enrichment of key metabolites in a separate MS experiment. The labelled cells containing a fully-labelled version of each metabolite present under the conditions tested will then be co-extracted with the systems under study or a previously made fully labelled extract will be spiked into the extraction. The ratio between the fully labelled and the monoisotopic mass peak for the unlabelled compound is determined in the MS experiment. This can be used directly to compare fold changes in metabolites under different experimental conditions as a relative quantification or, when the fully labelled extract is used as the matrix for the unlabelled external standards, quantitative data can be obtained [17,18].

This method has several drawbacks. Firstly, it is sometimes difficult to create the fully labelled extract. For example, organisms carrying auxotrophies [11] have broad carbon substrate requirements that may not be economically feasible under fully-labelled conditions (for instance, the cost of fully labelled amino acids easily increases to >$US 2000 per gram compared to $US 50–70 per gram for glucose). An alternative is to use a more common organism that can grow exclusively on glucose (e.g., *E. coli* or baker’s yeast), but this leads to the second and third problems. Such alternative organisms might not contain the entire metabolite pool present in the target organism, resulting in the labelled internal standard being missing for some compounds. The third problem is the low dynamic range of LC/MS. It is very difficult to achieve that both sample and internal standard are within the calibration curve. If different growth conditions or mutants are compared, it is likely that key metabolite pools will change significantly. This means that the sample would require different dilution than the internal reference. Another problem is the reproducible creation of internal standard mixes over long experimental campaigns. This will require the cultivation of the microbes in highly-defined systems, usually in chemostat mode.

## 3. Thermodynamics and Metabolomics Integration into Metabolic Networks

Metabolomic datasets are prone to errors and generally far from complete. Thermodynamics combined with the metabolic network structure can be employed to validate and expand metabolomic datasets. There are two main approaches to accomplish this: network embedded thermodynamic analysis (NET analysis) [19] and thermodynamics-based metabolic flux analysis (TMFA) [20]. NET analysis determines the feasible ranges of Gibbs free energy of reactions (Δ_r_G) for a given network and a given set of measured metabolite concentrations, and further determines the feasible concentration ranges for the unmeasured metabolites. Apart from enabling data validation (existence of a feasible solution), NET analysis can be used to determine metabolite distributions in compartmentalized models from total cell concentration. This can be especially important when dealing with compartmentalized microorganisms such as baker’s yeast. Since it is experimentally challenging to extract metabolites from separate compartments independently, to the best of our knowledge, there is only one publication that has been able to measure compartment specific metabolites concentration, where cytosolic and mitochondrial CHO cells metabolites were quantified [21]. TMFA, which uses a similar approach, focuses on finding thermodynamically feasible flux distributions by exploiting the directional constraints on reactions for which the feasible Δ_r_G range is either strictly negative or strictly positive. Thermodynamic analysis has been successfully applied to some microbial metabolic models, e.g., *Escherichia coli* [19,20,22], *Saccharomyces cerevisiae* [19,23], and *Geobacter sulfurreducens* [24], more recently the method has also being applied to a human model [23,25]

### 3.1. Second Law of Thermodynamics and Reactions Directionality

A system in thermodynamics is defined as a part of the universe that is of interest which, in our case, is the cell. The remainder of the universe is referred to as the surroundings. A system is classified as either closed or open, depending on whether it can exchange matter and energy with its surroundings. Thus, cells are open systems since they require external energy and nutrients, and release products into their surroundings.

There are two fundamental laws of thermodynamics:

- The *first law* is the conservation law which states that energy can neither be created nor destroyed. In a closed system energy is constant.
- The *second law* states that spontaneous natural processes increase the overall entropy of the universe.

The criterion of spontaneity is difficult to grasp because the entropy of the universe cannot be measured. For biological systems, where constant temperature and pressure apply, spontaneity is defined by the Gibbs free energy (ΔG ≤ 0). For a generic reaction (Equation (1)), the Gibbs free energy of the reaction (Δ_r_G) can be estimated by Equation (2); where R is the ideal gas constant, T the temperature and [I]^k^ the concentration of reactant I at the power of its stoichiometric coefficient k. Δ_r_G determines the directionality of a reaction, i.e., the net flux of the reaction occurs in the direction where Δ_r_G is negative.

(1) aA+bB→cC+dD,

(2) $$\Delta_{r}G_{j}^{\prime }=\Delta_{r}G_{j}^{\prime 0}+\mathrm{RTln}\left(\frac{{\left[C\right]}^{c}{\left[D\right]}^{d}}{{\left[A\right]}^{a}{\left[B\right]}^{b}}\right),$$

(3) $$\Delta_{r}G_{j}^{\prime 0}=\sum^{}S_{\mathrm{ij}}\Delta_{f}G_{i}^{\prime 0},$$

Δ_r_G depends on metabolites’ concentration (in the example reaction: A, B, C, and D) and the standard Gibbs energy of reaction (Δ_r_G^0^); which finally depends on the standard Gibbs energy of formation of the metabolites (Δ_f_G^0^) and the stoichiometric coefficients (represented by S_ij_, the stoichiometric coefficient of metabolite i in reaction j) (see Equation (3)). Thus, the directionality of reactions depends on the metabolites’ concentrations and their thermodynamic properties. The ‘'’ notation indicates physiological conditions are assumed rather than the standard biological conditions (which correspond to pH 7, zero ionic strength, 1 molar concentration in aqueous solution, 1 bar pressure, and 25 °C) [26]. It is important to consider these physiological conditions because it has been shown that pH and ionic strength significantly influence Δ_f_G [27,28].

### 3.2. Δ_f_G^0^ for Physiological Conditions

Δ_f_G^0^ represents Gibbs energy at standard conditions (denoted by the superscript ^0^). Intracellular conditions, however, are different from the standard conditions and it is essential to account for real cell conditions in terms of pH and ionic strength.

A more rigorous treatment when calculating the standard Gibbs energy of formation is to use activity (a_i_) instead of concentration (c_i_) of species. The activity coefficient (y_i_) correlates activity and concentration by the equation a_i_ = y_i_ × c_i_. To account for ionic strength, the extended equation of Debye-Hϋckel is utilized to calculate the activity coefficient:

(4) log(yi)=−AziI121+BI12,

where $z_{i}$ is the charge on ion  i, I is the ionic strength, A = 0.510651 L^1/2^·mol^1/2^, and B = 1.6 L^1/2^·mol^−1/2^ at standard pressure and temperature. The equation is valid within the ionic strength range of 0.005 to 0.25 M. The general equation to calculate the Gibbs energy of formation is expressed as:

(5) $$\Delta_{f}G_{i}=\Delta_{f}G_{i}^{0}+\mathrm{RTln}\left(a_{i}\right),$$

When the activity coefficient is considered:

(6) $$\Delta_{f}G_{i}=\Delta_{f}G_{i}^{0}+\mathrm{RTln}\left(y_{i}\right)+\mathrm{RTln}\left(c_{i}\right),$$

Equation (6) can be rewritten as:

(7) $$\Delta_{f}G_{i}=\Delta_{f}G_{i}^{0*}+\mathrm{RTln}\left(c_{i}\right),$$

where a new standard Gibbs energy of formation (Δ_f_G^0^*) is used, the notation * is employed here to remind the reader that this function accounts for ionic strength. Δ_f_G^0^* can be calculated using:

(8) ΔfGi0*=ΔfGi0−2.91482zi2I121+1.6I12,

For biochemical reactions, pH is assumed to be constant. What could be accomplished by the buffering capacity of intracellular proteins, so it is not necessary to balance hydrogen ions [26]. At constant pH and other than 7, the standard transformed Gibbs energy of species, i.e., the Legendre transform [29] of the standard Gibbs energy of formation is defined as:

(9) $$\Delta_{f}G_{i}^{\prime 0}=\Delta_{f}G_{i}^{0*}-N_{H}\Delta G\left(H^{+}\right)\;,$$

where N_H_ is the number of hydrogen atoms in the species. Substituting Equation (7) for the protons Gibbs energy yields:

(10) $$\Delta_{f}G_{i}^{\prime 0}=\Delta_{f}G_{i}^{0*}-N_{H}\left\{\Delta_{f}G^{0*}\left(H^{+}\right)+\mathrm{RTln}\left(10^{-\mathrm{pH}}\right)\right\},$$

Finally, the combination of Equations (8) and (10) produces an overall equation to calculate the standard transformed Gibbs energy of formation that accounts for physiological conditions (pH and ionic strength):

(11) ΔfGi′0=ΔfGi0−NH(i)RTln(10−pH)−2.91482(zi2−NH(i))I121+1.6I12,

### 3.3. Δ_f_G^0^ for Reactants as Groups of Species

Thermodynamics of biochemical reactions involves the study of reactions present in a living organism. From the point of view of thermodynamics, the main difference between biochemical and chemical reactions is that enzyme-catalyzed reactions are written in terms of reactants (e.g., ATP) that are made up of a sum of species (e.g., ATP4-, HATP3-, and MgATP2-) while chemical reactions are written in terms of species [26]. Therefore, it is necessary to account for all the species involved. To reduce the complexity of the calculations it is easier to work with reactants instead of individual species. The Gibbs energy of a reactant j is calculated by pseudo-isomers groups, whereby at chemical equilibrium all species (pseudo-isomers) have the same Gibbs free energy of formation, which is represented by Δ_f_G_j_; and the concentration of the reactant (isomer group) c_j_ is the sum of the concentration of species:

(12) $$c_{j}=\sum_{i=1}^{N}c_{i},$$

where N is the number of species i in the pseudo-isomer group. The Gibbs free energy of formation of the pseudo-isomer group is represented by:

(13) $$\Delta_{f}G_{j}=\Delta_{f}G_{j}^{\prime 0}+\mathrm{RTln}\left(c_{j}\right),$$

The Δ_f_G_i_ of the individual species at chemical equilibrium is given by:

(14) $$\Delta_{f}G_{i}=\Delta_{f}G_{i}^{\prime 0}+\mathrm{RTln}(c_{i}),$$

Finally using Equations (13) and (14) in Equation (12), and assuming that Δ_f_G_j_ = Δ_f_G_i_, the equation to calculate Δ_f_G_j_^0^ can be deduced [26]:

(15) $$\Delta_{f}G_{j}^{\prime 0}=-\mathrm{RTln}\left\{\sum_{i=1}^{N}\exp\left[-\frac{\Delta_{f}G_{i}^{\prime 0}}{\mathrm{RT}}\right]\right\},$$

The notation $\Delta_{f}G_{j}^{\prime 0}$ is used to show that it is a function of pH and ionic strength. It is important that the standard transformed Gibbs energy of formation of a reactant is not the sum of the standard transformed Gibbs energy of its species.

### 3.4. Gibbs Energy of Transport Reactions

A reaction that occurs between two compartments is considered a transport reaction; an example is the reaction of ATP synthase in the oxidative phosphorylation pathway, where protons are transported across the mitochondrial membrane (for eukaryotes) or the cell membrane (for prokaryotes). To calculate the Gibbs energy of a transport reaction, the reaction is first divided between the part that takes place in one compartment and the transmembrane transport portion, in order to separately calculate their Δ_r_G′^0^, and finally the Δ_r_G′^0^ ’s are added up [30]:

(16) $$\Delta_{r}G^{\prime 0}=\Delta_{r}G_{{\mathrm{comp}}_{i}}^{\prime 0}+\Delta_{r}G_{\mathrm{transport}}^{\;\prime 0}\;,$$

The calculation of Δ_r_G′^0^ of the transport term should take into account the pH and the membrane potential gradient between compartments. Under physiological conditions (pH ≠ 7 and ΔpH ≠ 0, ionic strength ≠ 0 and ∆Ψ ≠ 0 (the difference in membrane potential between compartments)), Δ_r_G′^0^ of transport is not zero and must be considered in the calculation of Δ_r_G′^0^ of a transport reaction. It is dependent on ∆Ψ and ∆pH [31]:

(17) $$\Delta_{r}G_{\mathrm{transport}}^{\prime 0}=\Delta_{\Delta \psi }G+\Delta_{\Delta \mathrm{pH}}G\;,$$

(18) $$\Delta_{\Delta \Psi }G\left(\mathrm{kJ}/\mathrm{mol}\right)=\mathrm{nF}\Delta \Psi \;,$$

(19) $$\;\Delta_{\Delta \mathrm{pH}}G\left(\mathrm{kJ}/\mathrm{mol}\right)=\overset{N}{\underset{i=1}{\sum }}s_{i}\Delta_{f}G_{j}^{\prime 0}-2.3\mathrm{RT}\overset{N}{\underset{i=1}{\sum }}s_{i}N_{H}\left(i\right){\mathrm{pH}}_{i},$$

where n is the net charge transported through the membrane, F is the Faraday constant, $\;s_{i}\;$ is the stoichiometric coefficient of the transported species i, and $\Delta_{f}G_{j}^{\prime 0}$ is the transformed Gibbs energy of transported metabolite j.

### 3.5. Example 1: Standard Transformed Gibbs Energies of Yeast Glycolysis Reactions at Physiological Conditions

To calculate the standard transformed Gibbs energy of reactions, Δ_r_G^′0^, it is necessary to account for the physiological conditions of yeast. These are assumed to be: cytosolic pH of 7 and internal ionic strength of 0.15 M [19]. Using these values and the charge, number of protons and Δ_f_G^0^ of the species involved in the reactions, Equations (11) and (15) can be employed to calculate the standard transformed energy of the compounds involved in the reactions (see Table 1). The Δ_f_G^0^ of NAD has been set to a reference value of zero and used to determine Δ_f_G^0^ of NADH.

The final Δ_r_G′^0^ values calculated for the standard transformed Gibbs energy for the reactions of glycolysis under physiological conditions in yeast are in Table 2. These can be calculated with Equation (3) using the values in Table 1. For example, the glucokinase (HEX1) reaction:

$$\begin{array}{ll} \Delta_{r}G_{\mathrm{HEX}1}^{\prime 0} & \\ & ={\;S}_{\mathrm{glucose},\mathrm{HEX}1}\Delta_{f}G_{\mathrm{glucose}}^{\prime 0}+S_{\mathrm{ATP},\mathrm{HEX}1}\Delta_{f}G_{\mathrm{ATP}}^{\prime 0}+S_{\mathrm{glucose}\;6P,\mathrm{HEX}1}\Delta_{f}G_{\mathrm{glucose}\;6P}^{\prime 0}+{\;S}_{\mathrm{ATDP},\mathrm{HEX}1}\Delta_{f}G_{\mathrm{ADP}}^{\prime 0}\\ & =(-1)*(-428.06)+(-1)*(-2292.28)+(1)*(-1319.75)+(1)*(-1425.17)\\ & =-24.58\mathrm{kJ}/\mathrm{mol} \end{array}$$

It is important to note that reactions in Table 2 are written without H^+^ because, as it has been explained in Section 3.2, pH is assumed to be constant (in this case at pH 7). On the other hand, the atoms of water are accounted for during the calculation of$\;\Delta_{r}G^{\prime 0}$.

In general, each reaction within a pathway is required to have a negative Δ_r_G for the pathway to proceed in the forward direction. Within an organism it is desired that, even allowing for variation in substrate and product concentration, the overall thermodynamic feasibility of a reaction is not affected. Therefore, typically the first and last reactions in a pathway have large negative Δ_r_G′^0^ to allow a very low substrate concentration and a high final product concentration without changing the thermodynamic feasibility of the pathway. This, in turn, means that the directionality of those reactions cannot be changed by manipulation of metabolite concentrations in a metabolic engineering exercise. In Table 2 it can be observed that reactions HEX1 and PYK have large negative Δ_r_G′^0^, illustrating the previous concept.

### 3.6. Application of Network Thermodynamics to Large-Scale Models

The application of network thermodynamics to large-scale models has three critical limitations: (1) lack of large-scale knowledge of network components; (2) lack of knowledge of thermodynamics properties (such as Δ_f_G^0^ and Δ_r_G^0^) of components at genome scale; and (3) lack of a structure to integrate the knowledge of different levels of biological networks [32].

In order to overcome the second limitation mentioned above, the so-called group contribution method is applied to estimate Δ_f_G^0^ and Δ_r_G^0^ [33,34]. Group contribution is a tool to increase the thermodynamic information due to the very limited availability of experimental data. For example, for the *E. coli* K-12 MG1655 genome-scale model (GeM) [35] experimental measurements of Δ_r_G^0^ are only available for 8.1% of the 2077 model reactions [36].

In order to estimate Δ_f_G^0^, this method divides each metabolite into groups. Each group represents a contribution to the final Gibbs energy (see Equation (20)), and can be estimated by linear regression, i.e.,

(20) $$\Delta_{f}G_{i}^{0}=P_{o}+\sum_{j}n_{\mathrm{ij}}P_{j}\;,$$

where $P_{o}$ is a constant contribution (called the origin), $P_{j}$ is the contribution of the jth group and $n_{\mathrm{ij}}$ is the number of times that the group j is in the compound i. In some cases extra contributions have to be added to account for other structural characteristics of the compound, such as aromatic rings and amide groups. This methodology has been recently updated using newly available experimental data to more accurately estimate group contributions and also includes new groups (e.g., molecules involving sulfur, nitrogen, and halogen structures) [36]. The updated method can determine the Δ_r_G^0^ of 93% of reactions in the KEGG database [37]. Pseudoisomeric group contribution is the latest extension of the group contribution method which, instead of decomposing compounds, it is based on groups for the pseudo-isomers (different protonation states) that made up the compounds. Therefore, it is able to estimate Gibbs energy with higher accuracy than group contribution for a larger range of pH conditions [38].

Recently a new approach that takes advantage of the larger coverage of pseudoisomeric group contribution method and the better accuracy of experimental data was developed to estimate Gibbs energies. The approach called component contribution method consistently combines both sources of Gibbs energy avoiding conflicts of reference point and violations of the first law of thermodynamics [39].

### 3.7. Example 2: Standard Gibbs Energy of Formation of Glucose Estimated by the Group Contribution Method

**Figure 2:**
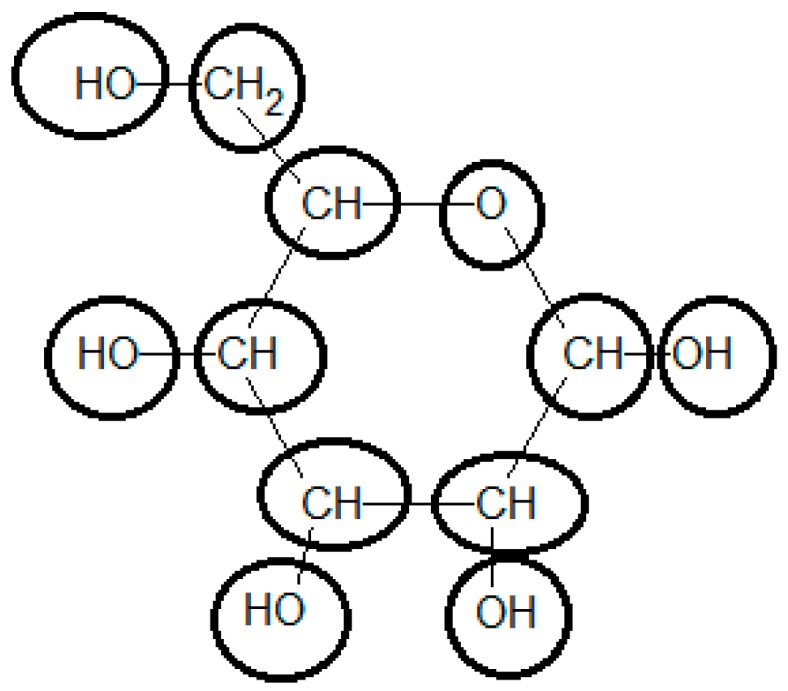
Decomposition of the chemical structure of glucose into groups to estimate its standard Gibbs energy of formation using a group contribution method.

Initially the chemical structure of glucose should be broken down into functional groups, using the most specialized possible groups. This is the most demanding task in the use of the group contribution method to determine the standard Gibbs energy of formation. To be able to use the methodology, the compound should be represented in its more common state in aqueous solution at standard conditions (i.e., pH = 7 and T = 25 °C) (see Figure 2). 

Glucose in ring form must be considered in the calculation of the final Δ_f_G^0^. Using the group contributions reported by Mavrovouniotis [33], the final value for Δ_f_G^0^ of glucose is −898.07 [kJ/mol], differing from the experimentally-observed value of −915.9 [kJ/mol] by 2% (see Table 3). Using the recently upgraded group contribution method [36] the Δ_f_G^0^ is estimated to be −913.90 [kJ/mol], which is substantially closer to the experimental value. Furthermore, the estimation of neutral glucose Δ_f_G^0^ by component contribution [39] is −916.3 [kJ/mol], even closer to the experimental value.

### 3.8. Incorporation of Quantitative Metabolite Data into Metabolic Networks

The first attempt to analyze thermodynamic feasibility of pathways instead of individual reactions was done by Mavrovouniotis, who also introduced the use of physiological conditions (Δ_r_G) instead of standard conditions (Δ_r_G^0^) [40].

To date, the range of Δ_r_G defined by physiologically feasible concentrations of metabolites has been widely applied to check for thermodynamic feasibility of reactions and pathways; as well as to evaluate reversibility of reactions and to possibly constrain the metabolite concentration range [19,20,22,30,41,42]. One interesting example is a study of the thermodynamic feasibility of biodegradation pathways [41]. Another interesting example is the creation of the metabolic GeM iHJ873 of *E. coli*, which incorporates thermodynamic properties of all network metabolites. It is based on the model iJR904 [43], but 85 compounds for which Δ_f_G have neither been determined experimentally nor are able to be estimated by the group contribution method have been eliminated. For this model a thermodynamic feasibility analysis of the reactions was achieved with new Δ_r_G^0^ values, considering all metabolites in concentrations of 1 mM instead of 1 M and, thus, being more consistent with physiological concentrations [30].

More recently, NET analysis was introduced [19], which applies thermodynamic principles to a GeM metabolic network to check for thermodynamic feasibility of metabolite datasets. NET analysis can also expand measured metabolite datasets by adding extra thermodynamically feasible metabolite concentration ranges that were not measured and can be used to predict putative regulatory sites. This algorithm incorporates the second law of thermodynamics and Δ_f_G′^0^ into a defined model. The optimization algorithm applied by NET analysis minimizes and maximizes Δ_r_G′ of each reaction in the network one by one (Equation (21)), resulting in the estimation of the $\Delta_{r}G_{k}^{\prime }$ range for all network reactions. The initially irreversible reactions (r_j_) in the network constrain their respective $\Delta_{r}G_{j}^{\prime }$ (represented by Equations (22) and (23)). The measured metabolite concentrations and the physiological concentration range add more constraints into the optimization approach (expressed in Equation (25)); also, the range of redox cofactor ratios can be incorporated as constraints. The final optimization is expressed as:

(21) $$\min/\max\;\Delta_{r}G_{k}^{\prime },$$

(22) $$s.t.\;\Delta_{r}G_{j}^{\prime }<0\;\forall \;r_{j}>0,$$

(23) $$\Delta_{r}G_{j}^{\prime }>0\;\forall \;r_{j}<0,$$

(24) $$\Delta_{r}G_{j}^{\prime }=\sum_{i}S_{\mathrm{ij}}\Delta_{f}G_{i}^{\prime },$$

(13) $$\Delta_{f}G_{i}^{\prime }=\Delta_{f}G_{i}^{\prime o}+\mathrm{RTln}\left(c_{i}\right),$$

(25) $$c_{i}^{\min}\leq c_{i}\leq c_{i}^{\max},$$

Equations (13) and (24) describe the calculation of$\;\Delta_{r}\;G\;\prime$. By this optimization the putative regulatory sites are calculated, these are reactions whose $\Delta_{r}\;G\;\prime$ is far from zero (reactions far from thermodynamic equilibrium are generally defined by Δ_r_G′_max_ < −10 [kJ/mol]), and are likely to be genetically or allosterically regulated [19]. In an analogous process, by changing the optimization objective to metabolite concentration, the range of all metabolite concentrations is calculated. The method was applied to *E. coli* and *S. cerevisiae*. Seven existing *E. coli* metabolite datasets were checked for consistency and only four were found to be thermodynamically feasible. For *S. cerevisiae*, it was shown that compound concentrations in the different compartments of the cell can be estimated from average cell concentrations. The MATLAB software *anNET* implements NET analysis on a user-friendly platform and is freely available for academic use [44]. *NExT*, an open-source MATLAB implementation of NET analysis was later developed to correct calculations of Gibbs energy of transport reactions [45]. Thermodynamics-based metabolic flux analysis (TMFA) goes one step further, estimating metabolic flux distributions utilizing thermodynamic constraints directly by metabolic flux analysis (MFA) [20]. The result is a thermodynamically feasible flux profile. The method has been applied to *E. coli* [20] and *Geobacter sulfurreducens* [24]. For *E. coli*, putative regulatory sites were calculated and it was shown that 30 out of 86 of these sites are the first steps in their respective pathways.

Another approach to estimate flux distribution and metabolite concentration is to direct and simultaneously incorporate mass balance and thermodynamic constraints in a single optimization problem [46]. The advantage of this approach is the incorporation of measured metabolite datasets into the search for feasible flux distributions. However, the complex optimization objective, which simultaneously minimizes or maximizes the flux solution and the difference between the measured metabolite concentrations and the thermodynamically feasible concentrations, makes the algorithm cumbersome and slow.

For all of the algorithms described above, the focus is on the calculation of feasible metabolite concentrations and/or flux distributions using known reaction directionalities as constraints. The idea of using metabolomics data and thermodynamic constraints to assign reaction directionality in a metabolic network was recently reported by Fleming and co-workers [22]. In this work, the physiological ranges of metabolite concentrations were used in the iAF1260 *E. coli* metabolic model [35] to determine the directions of reversible reactions by calculating minimum and maximum of Δ_r_G of each reaction in the network individually. A priori, all reactions were considered reversible [22]. They found that the joint use of the experimentally determined and the estimated (by group contribution method) Δ_r_G′^0^ increased the scope and accuracy of the predictions. The previous approach individually assigns directions to initially reversible reactions without taking into account the network context. When the network topology is considered, individual irreversible reactions could cause another reaction to become irreversible through metabolites concentration constraints, therefore a wide propagation of the thermodynamic constrains may take place. NET analysis, despite of initially being developed to thermodynamically validate metabolomics data; it is possible to use it in reverse to assign directionalities based on metabolites’ concentration ranges [23]. This approach was used to perform a network thermodynamically curation of a yeast and a human metabolic model, Yeast 5 and Recon 1, respectively. In addition to thermodynamically assigning and validating directionalities of the network reactions, some interesting biological insights were found: (1) the use of different mechanisms by different organisms to overcome unfavorable thermodynamics of the phosphoribosylaminoimidazole carboxylase reaction; and (2) the different compartmentalization of proline metabolism in yeast cells and humans [23].

The reader is referred to Atam’s review [47] for further information on the latest publications on network thermodynamic analysis. In this review the reduction of biologically relevant elementary flux modes (EFMs) by a thermodynamic feasibility analysis is presented as one of the latest applications of networks thermodynamics. EFMs represent the network metabolic capabilities, however, the enumeration of EFMs is a cumbersome problem, thus, the reduction of EFMs to biologically relevant ones is an extremely useful approach. The first attempt of thermodynamic analysis of EFMs was realized to an *E. coli* metabolic network [48]. Later, a full NET analysis of EFMs was performed for a yeast model [49]. The latest approach is tEFMA, a software that, while enumerates the EFMs is checking for thermodynamics feasibility in order to reduce the time in the EFMs generation [50,51].

### 3.9. Non-Equilibrium Thermodynamics for Enzymatic Reactions

Cellular systems are open, which share mass and energy with their surroundings outside, and they have many internal processes that operate far from equilibrium. At thermodynamic equilibrium the flow through cellular pathways would be zero and the cell would stop functioning. A good example is the process of passive diffusion, where a concentration gradient is required (driving force).

The second law of classical thermodynamics together with the Gibbs energy of reactions can be used to determine the feasibility of metabolite concentrations and directions of reactions; however, it does not provide any information about the reaction rates. Non-equilibrium thermodynamics studies the relationship between flow and thermodynamic driving force, known as the flow-force relationship. This relationship state that flux depends on both capacity and driving force (Equation (26)); for example, the flow of water in a tube is a function of the diametric area of the tube (which is called capacity) and the energy that is moving the water. The relationship can be expressed as:

(26) Flow≈capacity × force ,

In the specific case of enzymatic reactions the metabolic flux is a function of the enzyme activity (capacity) and thermodynamic driving force (force):

(27) Flux ≈ enzyme activity × thermodynamic driving force ,

The thermodynamic driving force in non-equilibrium thermodynamic is called affinity, A, which is equal to −Δ_r_G (function of metabolomics). At near equilibrium with excess capacity, there is a proportional relation between flux (v) and affinity (A):

(28) v=LA,

where L is the phenomenological coefficient that represents the enzyme capacity (enzyme activity) [52]. In other words, a doubling in affinity should lead to a doubling in flux. This relationship has been demonstrated to apply across a fairly broad flux range of non-equilibrium reversible reactions when there is mass conservation [53]. In the case of non-equilibrium irreversible reactions there is an offset and the relationship is linear rather than proportional [53,54]. It remains true, however, that reactions under thermodynamic driving force, as opposed to capacity control, will be characterized by an increase in affinity with increased flux.

The previous concept can be illustrated by the example of a simple reversible unimolecular reaction (S → P). At steady state, and when the sum of substrate (Cs) and product (Cp) concentrations is kept constant (i.e., Cp + Cs = C), the net rate can be expressed in terms of affinity [52,54]:

(29) vvsmax=exp(ART)−1((KsC)+1)exp(ART)+(vsmaxvpmax)((KpC)+1),

where $v_{\mathrm{smax}}$ and $v_{\mathrm{pmax}}$ are the maximum forward and backward reaction rates, respectively, and $K_{s}$ and $K_{p}$ are the Michaelis–Menten constants.

**Figure 3:**
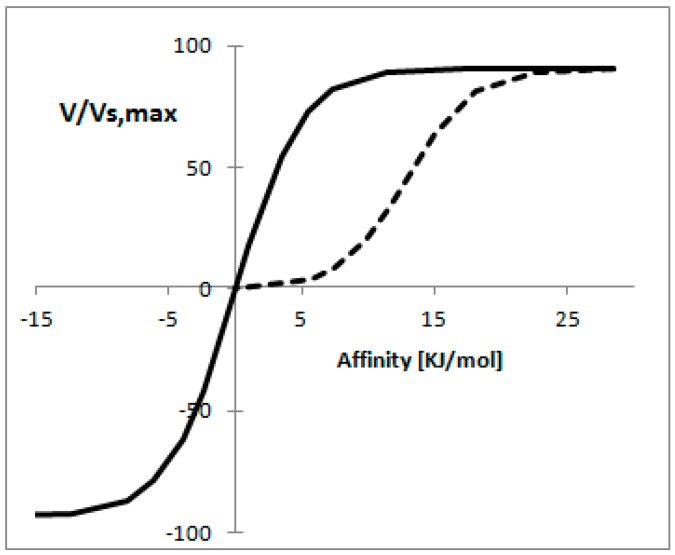
Forward reaction rate (v) of an enzyme-catalyzed reaction as a function of Affinity. The total concentration of substrate and product is assumed to be constant and equal to 10 mM. In solid line is represented a thermodynamic reversible reaction (v_p_ = 100 [mmol/min mg], K_p_ = K_s_ = 1) and the dashed line represents a thermodynamically irreversible reaction (v_p_ = 1, K_p_ = 10 and K_s_ = 1).

Figure 3 shows the reaction rate as a function of affinity when the concept of mass conservation applies, from this figure is clear that for the thermodynamically reversible reaction a proportional relationship is found between the reaction rate and affinity for a wide range of reaction rates. In the case of the thermodynamically irreversible reaction it is possible to observe a linear relationship [55].

The strongest assumption made in the previous analysis is that C is kept constant. While this assumption is generally valid, it has been shown that this is not the case for some pathways. In such cases the concentrations have other physical constraints. For example, Rottenberg [53] showed that if the concentration of substrate or product was kept constant, the relationship between the reaction rate and affinity was nearly linear [53].

A generalized approach for kinetic approximation of enzymatically catalyzed reactions was subsequently developed, namely linlog kinetics [56]. This approximation is based on the previous described thermodynamic principles, but also considers the presence of allosteric effectors by including them with logarithmic concentrations in the equation. However, because there are currently few in vivo measurements of regulator concentrations, in practice this remains only a good theory, which has the potential to become useful with improvements in measurement methods.

## 4. Quantification of Metabolic Phenotype Using ^13^C Fluxomics

^13^C fluxomics provides a measure of the metabolic phenotype, namely the in vivo enzyme activity measured as the molar flux through each reaction in the network. Only the most peripheral fluxes, such as uptake and secretion rates, can be measured directly with quantitative metabolite analysis, while fluxes within a metabolic network are inferred from stoichiometric models. Four major approaches for metabolic flux analysis have been developed over the last two decades: kinetic modeling [57], flux balance analysis (FBA) [58,59], dynamic flux modeling [60,61], and ^13^C metabolic flux analysis (^13^C-MFA) [62].

As we have seen in the case of non-equilibrium thermodynamics, kinetic modelling relies on knowledge of the kinetic parameters of all enzymes under study. These parameters are usually very hard, or even impossible, to determine in vivo and, therefore, the application of kinetic modelling currently remains limited to small local networks.

FBA, also commonly called metabolic flux analysis (MFA), allows the calculation of net-fluxes in the cell using mass balancing, which means that the overall sum of all molar fluxes entering and leaving a metabolite pool must be zero. It can be applied to underdetermined networks with the use of constraints (e.g., reversibility of reactions, upper and lower boundaries of rates, etc.), the inclusion of co-factor balances and/or the application of an optimization function (e.g., to maximize growth). While it fails to resolve parallel pathways [63] and reversibility of enzymes [64,65], it is applicable to very large networks (>500 reactions), such as genome-scale models. Most importantly, it can be used in a predictive way to study “what if” scenarios prior to any lab work; for example, gene essentiality by *in silico* gene knockout. Dynamic flux modeling is an extension of MFA, where the same approach is used to estimate internal metabolic fluxes relying on external metabolomics data, but in this case is metabolomics data over-time, therefore, fluxes over-time are estimated. The approach is useful to describe fluxes of transition states. However, similarly to conventional MFA, it is unable to estimate fluxes for parallel pathways and reversibility of enzymes.

^13^C-MFA is another extension of FBA. In this approach, precursors enriched in the stable carbon isotope ^13^C are introduced into the metabolic system under study. Metabolism subsequently redistributes the labelled carbon atoms. The level of ^13^C enrichment in metabolites is measured via MS or NMR spectroscopy. These enrichments are used together with stoichiometric constraints of the network to quantify intracellular metabolic fluxes. This enables resolution of parallel pathways and reversibility of enzymes. In contrast to traditional FBA, co-factor balances are not used as constraints for flux calculations, but can be inferred from flux results. Typically, ^13^C-MFA focuses on central carbon metabolism delivering a flux estimate that describes the metabolic state of a cell at a given time. There are several detailed reviews of ^13^C-MFA [66,67,68,69].

^13^C-MFA was first developed for bacteria [70] and yeasts [71] and has predominantly been used for the study of microbial growth in a defined medium on a single carbon source, which greatly simplifies determination of uptake and secretion rates, closure of mass balances and labelling analysis of target metabolites. In recent years, ^13^C-MFA has been combined with other omics techniques for in-depth characterization of microbial systems [72,73]. This combination of techniques provides a deeper understanding of the underlying regulation and its uses have been diverse, e.g., metabolic engineering, drug design, or basic biological research. With the development of more sophisticated quantitative metabolomics methods and the availability of faster modelling algorithms, ^13^C-MFA will be more readily applicable to more complex systems.

^13^C-MFA relies on the assumption that the system is at a pseudo-steady state, e.g., the molar flux through a metabolite pool is orders of magnitude larger than changes in concentration of this metabolite over time, so that changes in concentrations do not significantly affect the fluxes. Thus, the rate of change in metabolite concentration, c, over time is assumed to be nil. Mathematically, this is expressed as:

(30) $$\frac{\mathrm{dc}}{\mathrm{dt}}=\mathrm{Sv}=0,$$

where S and v denote the stoichiometric matrix and the flux vector, respectively.

The pseudo-steady state assumption is generally valid for continuous cultures and during unlimited exponential growth. Exceptions are extreme transient states or accumulation of compatible solutes in the system. In addition, it is assumed that there is no isotope effect, meaning that enzymes do not have a significant preference for labelled or non-labelled substrates and that the system is well mixed. Moreover, recent publications have shown that the isotope effect, if present, has a negligible impact on metabolism [74,75]. Simulation is easier if the system under study is also at its isotopic steady state, e.g., the labelling distributions of the metabolites are stable. Consequently, it depends on the pool size of metabolites and the fluxes through the pool determine how long it takes for the labelling distribution in the pool to reach the isotopic steady state. For free intracellular amino acids, for example, this can be reached within minutes to hours [73], while proteinogenic amino acids derived from protein hydrolyzates will take several cell doublings before the isotopic steady state is reached.

For a more dynamic analysis, free intracellular pools of low abundant metabolites are ideal (e.g., sugar phosphates in glycolysis) because the isotopic steady state is quickly achieved and the measurement of enrichment kinetics is unnecessary.

### 4.1. Performing a ^13^C Metabolic Flux Analysis

**Figure 4:**
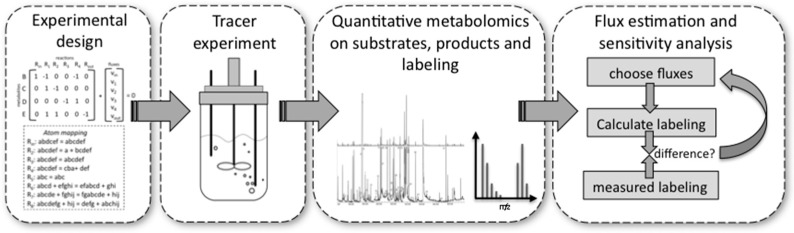
Workflow of a typical ^13^C metabolic flux analysis experiment.

^13^C-MFA can be separated into four different phases (Figure 4).

1. *Experimental design* is important because very costly tracer substrates can be ineffective when used in a suboptimal design. For design, the stoichiometry and the atom transitions must be known. By performing labelling experiments *in silico* for the expected range of fluxes, it is possible to determine the most suitable labelled substrate(s) to use and the most suitable labelled metabolites to analyse by MS or NMR, e.g., the metabolites for which labelling patterns are most responsive to changes in fluxes. These metabolites can be derived from macromolecules, such as proteinogenic amino acids or free intracellular metabolites. The choice depends on available sample size and equipment sensitivities.
2. *Performing the experiment:* Pseudo-steady state conditions require balanced growth throughout the tracer experiment. It is essential that no nutrient limitations, for example oxygen in aerobic experiments, occur during the experiment. Biomass composition should remain fairly constant and the mass balances of the system for at least one macro-nutrient, for example carbon or nitrogen, should be closed. Bioreactors guarantee such a controlled environment with balanced growth, but it has been shown that shake flasks and microplate systems can also deliver these conditions within certain limits [76].
3. *Quantitative metabolomics:* All major substrates and products need to be accurately quantified. For MFA the biomass itself is an important product. Its major composition (DNA/RNA, protein, carbohydrate, lipid contents) determines a whole range of anabolic fluxes. Finally, MS [77] or NMR [70] equipment are required to quantify the labelling enrichment in the target metabolites (e.g., GC/MS analysis of proteinogenic amino acids). Accurate isotope ratios, although not absolute quantification, of those metabolites is essential.
4. *Flux estimation and sensitivity analysis:* Flux estimation is an iterative process in which the stoichiometric and atom mapping networks are used to calculate labelling outputs while achieving the experimentally observed uptake, secretion, and growth rates. At each iteration the calculated labelling outputs are compared to the experimentally determined ones and the resulting fluxes are updated until the differences between calculated and measured labellings are minimized. At the end of this fitting process, the sensitivity of fluxes is further evaluated using statistical procedures. As a result, each flux is obtained with a certain confidence interval.

While phases 2 and 3 demand sophisticated experimental and analytical procedures, phases 1 and 4 require sophisticated modelling software. We recently implemented one of the fastest algorithms for ^13^C-MFA, the EMU (Elementary metabolite unit) approach [78], in the easy to use, open source software package called OpenFLUX [79] (https://sourceforge.net/projects/openflux/). This software can be used for experimental design, as well as for flux estimation and sensitivity analysis. It currently supports the most common platform for labelling analysis, mass spectrometry, but because it is open source users can include their own code in order to use NMR fine spectral information. It also supplies the atom transitions for common reactions of central carbon metabolism, but can be custom designed for any network. In addition to OpenFLUX there are several software packages available to facilitate steady state isotope ^13^C flux analysis, the most popular are: FiatFlux [80], Metran [81], Isodesing [82], and 13CFLUX2 [83], an updated version of 13CFLUX [69]. The different packages differ in the complexity of inputs required, the data processing method, precision of results, computational time, friendliness of the user interface, possibility to modify source code, software language, and flexibility. Recently new software packages, INCA [84] and OpenMebius [85], were developed to perform isotopically non-stationary ^13^C metabolic flux analysis.

### 4.2. Example 3: Integration of Quantitative Metabolite Data, Thermodynamics and ^13^C Fluxomics Based on Saccharomyces Cerevisiae GeM

*Saccharomyces cerevisiae* S288C was cultured on a chemically defined medium containing glucose and glutamate as carbon and nitrogen sources. Aerobic cultivation was performed in a bioreactor at pH 6.0 and 30 °C in continuous mode at a measured dilution rate of 0.29 [h^−1^]. Samples were taken during steady state after five residence times.

Quenching was performed as described in [86] and extraction was done in boiling water containing internal standards.

Extracellular glucose, ethanol, glycerol, and organic acids, as well as intra- and extracellular amino acids, were quantified using HPLC as described by Dietmair et al. [10]. Analysis of endo-metabolites by LC-ESI-MS/MS was performed on an ABSciex 4000 QTRAP mass spectrometer (ABSciex, Concord, Canada) coupled to a HPLC instrument. Using an ion-pairing method adapted from Luo et al. [14]. In order to reduce the influence of matrix effects, the standards were prepared in a fully ^13^C-labelled yeast extract, obtained from a continuous culture on universally labelled (U^13^C) glucose at a dilution rate of 0.2.

In addition, a separate tracer experiment for ^13^C flux analysis under identical conditions was performed. This was necessary since the ^13^C tracer alters the mass distributions of target metabolites, leading to mismatches with the MRM’s set up in the LC-ESI-MS/MS analysis. The physiological match between the tracer and non-tracer experiment was ensured using HPLC and growth data. A 20:80 mix of 99% [1-^13^C] glucose and 99% [U-^13^C] glucose was used as the labelled substrate together with naturally-distributed glutamate. Cell hydrolysis and labelling analysis are described elsewhere [72,87].

To apply NET analysis the yeast consensus GeM, Yeast 5 [88] was modified as previously described [23], in order to be able to use thermodynamics the following changes of the model were needed [23]:

- reactions catalyzed by different enzymes in either direction should be lumped together resulting in a reversible reaction, otherwise a thermodynamic equilibrium state with a zero Gibbs energy of reaction will be considered for both of them;
- in thermodynamics, reactions are described in terms of reactants instead of species. For example the species HCO^3−^, CO_3_^2−^, CO_2_, and H_2_CO_3_ are grouped as the reactant CO_2tot_ because aqueous phase carbon dioxide is distributed in those species;
- for reactions that contain CO_2tot_, H_2_O was added to the opposite side of the reaction in order to balance oxygen atoms; and
- the oxidized and reduced flavin adenine dinucleotide (FAD) in mitochondria were replaced by oxidized and reduced FADenz in order to represent the enzyme-bound FAD cofactor.

Based on the same GeM, an EMU model for ^13^C flux estimation was created in OpenFlux [79]. The model contained 406 metabolic reactions. Flux analysis was performed using the stoichiometric rates for substrate uptake, product formation and anabolism alongside the labelling enrichment data of 18 mass distribution vectors from proteinogenic amino acids as determined by GC/MS. The concentration range for each metabolite and a range of Δ_r_G for each reaction were estimated using *NExT* [45]. Initially, broad concentration ranges were used in order to check the model’s thermodynamic feasibility.

**Figure 5:**
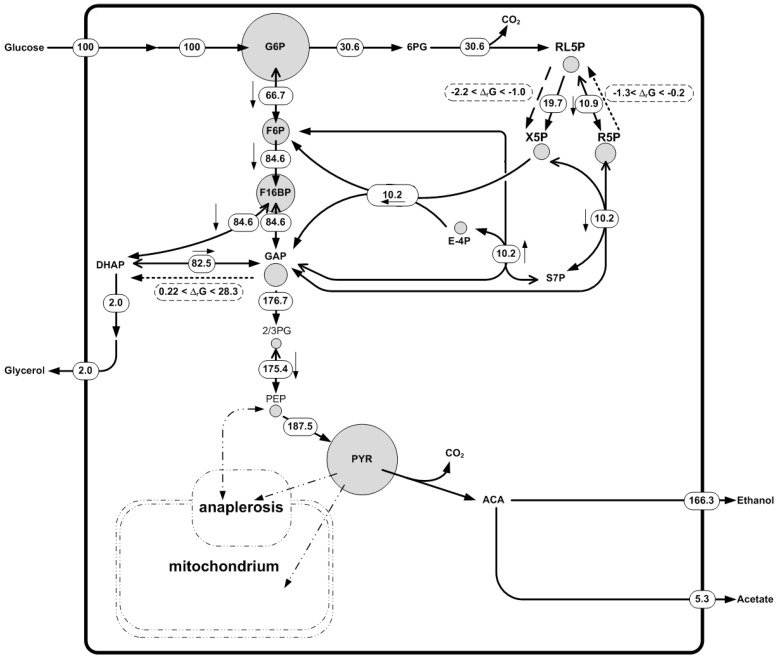
Intracellular metabolite concentrations and metabolic fluxes in *Saccharomyces cerevisiae* S288C during growth on glucose and glutamate as carbon sources in continuous culture. The dilution rate is 0.29. Metabolite concentrations are represented as circles corresponding to their concentration in [μmol/g_CDW_]. Concentrations were set equal to mm^2^. After back-calculating to the radius of the circle, the 10-fold radius was used to create the circles in the figure. Net fluxes are presented. In the case of reversible reactions, depicted by a double arrow, the net direction is indicated with a small arrow next to the flux value. Δ_r_G values were predicted by using *NExT*. Dashed lines indicate flux direction based on thermodynamic modelling. Dotted lines show the predicted flux direction by the thermodynamic modelling contradicted by ^13^C fluxomics where Δ_r_G is close to zero. For simplification, only a small section of the network is shown and for clarity some fluxes are omitted. Each metabolite node is balanced in the full network.

Subsequently, metabolite data of the *Saccharomyces cerevisiae* fermentation was used. Concentrations of amino acids, nucleotides, and some metabolites of the central carbon metabolism were measured. A broad concentration range was assigned to non-measured metabolites. The measured metabolite concentration dataset was found to be thermodynamically feasible. Using this dataset we were able to predict a new irreversible reaction in the pentose phosphate pathway (Figure 5), namely d-ribulose-5-phosphate 3-epimerase. The reaction was shown to operate in the direction of production of Xylulose-5-phosphate with Δ_r_G in the range of −2.2 to −1.0 [kJ/mol]. ^13^C fluxomics independently confirmed that the net flux of this reaction was in the direction the metabolite data and the thermodynamics predicted (Figure 5). Two other reversible reactions in the network were predicted to be irreversible: d-ribose-5-phosphate ketol-isomerase and triose-P-isomerase. In these cases the Δ_r_G was between −1.3 and −0.2 (kJ/mol) and 0.22–28.32 [kJ/mol], respectively (Figure 5). The predicted directionality contradicted the observed net fluxes in the ^13^C fluxomics. This highlights the fact that a Δ_r_G range close to equilibrium (Δ_r_G = 0) meaning that the reaction is likely to be reversible. In fact, the ^13^C fluxomics also showed a high reversibility for these reactions. There was no measurement of di-hydroxy acetone phosphate (DHAP) available, which may help to fix the directionality of the triose-*P*-isomerase reaction, underlining the importance of complete metabolite datasets.

In summary, this example shows how the different datasets are complementary to one another and can be used to gain deeper insights into metabolism and higher confidence in the results. Thermodynamics is useful to check the quality of the metabolomics data and ^13^C fluxomics to describe the metabolic phenotype. Moreover, when opposing information on reactions directionality was found between the methods, the reactions are close to equilibrium and should be described as reversible. If it is not possible to estimate metabolic fluxes with ^13^C fluxomics due to the impossibility to reach metabolic pseudo-steady state or the feeding with labeling substrate is too expensive, thermodynamic-based network analysis is presented as a powerful tool to determine a reactions’ directionality, information that can be used with conventional MFA to determine a reduced solution space for the metabolism of the studied organism.

## 5. Conclusions

Despite a great increase in sensitivity, selectivity, and coverage of the platforms to measure metabolomics, it is still not possible to generate a full organism’s quantitative metabolomics data. Furthermore, when ^13^C-labelled metabolites are required the availability of standards become an issue due to the high price of these labelled metabolites. An approach recently applied to overcome this issue is the use of labelled internal standards generated by culturing microbes with labelled carbon substrates. However, the generated labelled metabolites will depend on the used organism and the reproducibility is highly dependent on the standardization of the culture conditions. Therefore, there are still challenges that need to be overcome in order to increase metabolomics data quality.

The introduction of the second law of thermodynamics into a metabolic network will determine directionality of initially-reversible reactions, constraining the solution space of metabolic networks using experimental data and thermodynamics principles. With the increase on coverage an accuracy of Gibbs energy determination is now possible to include thermodynamics constraints into metabolic flux determinations. The determination of compartment/organelle specific metabolite concentrations is in its infancy, presenting an extra challenge when working with compartmentalized models, such us yeast and humans. Despite NET analysis being able to estimate compartment-specific ranges of metabolites concentrations from average cell metabolites concentrations, the higher coverage of compartment specific metabolomic data will increase the power of network thermodynamics approaches.

Here an example was presented on how quantitative metabolomics data: endo- and extra-metabolites’ concentrations and enrichment level of isotopically-labelled metabolites are used to determine an organism metabolic phenotype. Two different approaches were employed: ^13^C fluxomics and network thermodynamics analysis. It was shown that both approaches complement each other. However, some discrepancies were found which are mainly explained by the need of; (1) more accurate Gibbs energy or known errors of the Gibbs energy values; and (2) higher coverage of endo-metabolomics data. Moreover, other omics data, such as proteomics and transcriptomics, could be used in combination with the previous technologies to provide a deeper biological understanding of the organism metabolic phenotype and regulation.

## Figures and Tables

**Table 1:** Standard transformed Gibbs energy for metabolites involved in glycolysis.

Metabolite	Δ_f_G^0^ [kJ/mol]	Charge (z_i_)	Number of H Atoms (N_H_)	Δ_f_G′^0^ [kJ/mol]
3-Phospho-glyceroyl phosphate	−2356.14	−4	4	−2206.35
	−2401.58	−3	5	
2-Phospho-glycerate	−1496.38	−3	4	−1341.51
	−1539.99	−2	5	
3-Phospho-glycerate	−1502.54	−3	4	−1347.41
	−1545.52	−2	5	
ADP	−1906.13	−3	12	−1425.17
	−1947.1	−2	13	
	−1971.98	−1	14	
ATP	−2768.1	−4	12	−2292.28
	−2811.48	−3	13	
	−2838.18	−2	14	
Glycerone phosphate	−1296.26	−2	5	−1095.82
	−1328.8	−1	6	
Fructose 6-phosphate	−1760.8	−2	11	−1316.55
	−1796.6	−1	12	
Fructose 1,6-bisphosphate	−2601.4	−4	10	−2206.14
	−2639.36	−3	11	
	−2673.89	−2	12	
Glyceraldehyde 3-phosphate	−1288.6	−2	5	−1088.16
	−1321.14	−1	6	
Glucose	−915.9	0	12	−428.06
Glucose 6-phosphate	−1763.94	−2	11	−1319.75
	−1800.59	−1	12	
H2O	−237.19	0	2	−155.88
NAD	0	−1	26	1056.29
NADH	22.65	−2	27	1117.50
Phosphoenolpyruvate	−1263.65	−3	2	−1189.04
	−1303.61	−2	3	
Pi	−1096.1	−2	1	−1059.30
	−1137.3	−1	2	
Pyruvate	−472.27	−1	3	−351.01

**Table 2:** Standard transformed Gibbs energy for reactions of glycolysis. HEX1: Hexokinase, PGI: Glucose 6-phosphate isomerase, PFK: Phosphofructokinase, FBA: Fructose-bisphosphate aldolase, TPI: Triose-phosphate isomerase, GAPD: Glyceraldehyde 3-phosphate dehydrogenase, PGK: Phosphoglycerate kinase, PGM: Phosphoglycerate mutase, ENO: Enolase, PYK: Pyruvate kinase.

rxnID	Extended Reaction	Δ_r_G′^0^ (kJ/mol)
HEX1	Glucose + ATP = Glucose 6-phosphate + ADP	−24.58
PGI	Glucose 6-phosphate = Fructose 6-phosphate	3.20
PFK	Fructose 6-phosphate + ATP = Fructose 1,6-bisphosphate + ADP	−22.48
FBA	Fructose 1,6-bisphosphate = Glycerone phosphate + Glyceraldehyde 3-phosphate	22.15
TPI	Glycerone phosphate = Glyceraldehyde 3-phosphate	7.66
GAPD	Glyceraldehyde 3-phosphate + Pi + NAD = NADH + 3-Phospho-glyceroyl phosphate	2.32
PGK	3-Phospho-glyceroyl phosphate + ADP = 3-Phospho-glycerate + ATP	8.16
PGM	3-Phospho-glycerate = 2-Phospho-glycerate	5.90
ENO	2-Phospho-glycerate = Phosphoenolpyruvate + H2O	−3.41
PYK	Phosphoenolpyruvate + ADP = Pyruvate + ATP	−29.08

**Table 3:** Calculation of the Δ_f_G^0^ of glucose by the group contribution method. OH-groups are distinguished according to their attachment to primary or secondary carbon atoms. Contributions of groups -O- and >CH- take into account their presence in a ring.

Group	Contribution ^1^ [kJ/mol]	Contribution ^2^ [kJ/mol]	# Occurrences	Total Contribution ^1^ [kJ/mol]	Total Contribution ^2^ [kJ/mol]
Ori	−103.4	0.0	1	−103.4	0.0
OH- (secondary)	−131.5	−173.8	4	−525.9	−695.0
-O- (ring)	−101.7	−153.2	1	−101.7	−153.2
>CH_2_	7.1	6.8	1	7.1	6.8
>CH- ( ring)	−10.9	20.3	5	−54.4	101.3
OH- (primary)	−119.7	−173.8	1	−119.7	−173.8
Total	-	-	-	−898.07	−913.89

^1^ Contributions from Mavrovouniotis et al. [33]; ^2^ Contributions from Jankowski et al. [36].
